# B cell metabolism in autoimmune diseases: signaling pathways and interventions

**DOI:** 10.3389/fimmu.2023.1232820

**Published:** 2023-08-23

**Authors:** Jingyue Li, Mingjiu Zhao, Wenjun Luo, Jiaqi Huang, Bin Zhao, Zhiguang Zhou

**Affiliations:** ^1^ National Clinical Research Center for Metabolic Diseases, Metabolic Syndrome Research Center, Key Laboratory of Diabetes Immunology, Ministry of Education, Department of Metabolism and Endocrinology, The Second Xiangya Hospital of Central South University, Changsha, Hunan, China; ^2^ Xiangya School of Public Health, Central South University, Changsha, China; ^3^ Furong Laboratory, Central South University, Changsha, China

**Keywords:** autoimmune diseases, autoimmunity, B cell, B cell metabolism, B cell differentiation and function

## Abstract

Autoimmune diseases are heterogeneous disorders believed to stem from the immune system’s inability to distinguish between auto- and foreign- antigens. B lymphocytes serve a crucial role in humoral immunity as they generate antibodies and present antigens. Dysregulation of B cell function induce the onset of autoimmune disorders by generating autoantibodies and pro-inflammatory cytokines, resulting in an imbalance in immune regulation. New research in immunometabolism shows that cellular metabolism plays an essential role in controlling B lymphocytes immune reactions by providing the energy and substrates for B lymphocytes activation, differentiation, and function. However, dysregulated immunometabolism lead to autoimmune diseases by disrupting self-tolerance mechanisms. This review summarizes the latest research on metabolic reprogramming of B lymphocytes in autoimmune diseases, identifying crucial pathways and regulatory factors. Moreover, we consider the potential of metabolic interventions as a promising therapeutic strategy. Understanding the metabolic mechanisms of B cells brings us closer to developing novel therapies for autoimmune disorders.

## Introduction

Autoimmune disorders manifest when the immune system erroneously attacks the organs, tissues, or cells of the body (1). Typically, the crucial role of humoral immunity is to safeguard the body from external pathogens, ensuring a balanced state of homeostasis. However, in autoimmune disorders the immune system becomes dysregulated and attacks healthy tissue and cells, causing damage to the body (2). The incidence of autoimmune diseases is increasing globally, affecting around 5% of the world’s population, making it a major public health concern (3). Existing evidence indicates that the development of autoimmune diseases involves a multifaceted interaction among genetic predisposition, environmental risk factors (including infections, exposure to harmful chemicals, and smoking), and immune dysregulation. This intricate interplay ultimately brings about the defect of self-tolerance and the onset of these disorders (3, 4).

**Figure 1:**
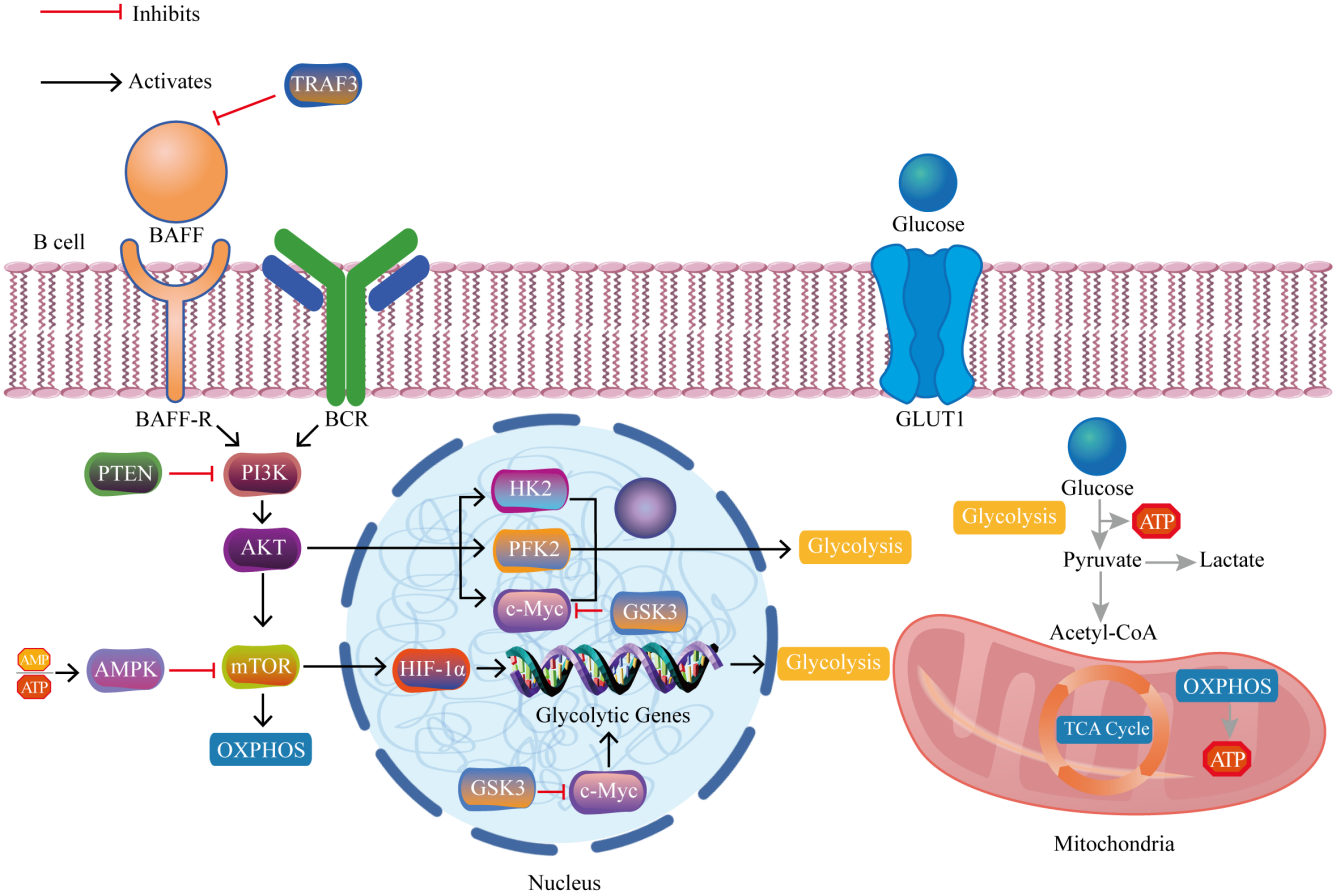
The crucial pathways and regulatory molecules of B cell metabolic reprogramming in autoimmune diseases. The interaction between BAFF and its receptor BAFF-R on B cell membranes triggers activation of the PI3K/AKT/mTOR/HIF-1α pathway. HIF-1α, together with c-Myc, upregulates genes associated with glycolysis, thereby promoting glucose uptake and glycolytic metabolism. TRAF3 inhibits excessive BAFF expression and its activation-related downstream cascades. PTEN inhibits PI3K and its activation-related downstream cascades. AMPK inhibits mTOR and its activation-related downstream cascades. GSK3 inhibits c-Myc and its activation-related downstream cascades. BAFF, B cell activating factor; BAFF-R, B cell activating factor receptor; BCR, B cell receptor; PI3K, phosphoinositide 3-kinase; AKT, protein kinase B; mTOR, mammalian target of rapamycin complex; HIF-1α, hypoxia-inducible factor 1-alpha; c-Myc, cellular myelocytomatosis oncogene; TRAF3, tumor necrosis factor receptor-associated factor 3; AMP, adenosine monophosphate; ATP, adenosine triphosphate; AMPK, AMP-activated protein kinase; PTEN, phosphatase and tensin homolog; GSK3, glycogen synthase kinase 3; HK2, hexokinase 2; PFK2, phosphofructokinase 2; GLUT1, glucose transport 1; TCA cycle, tricarboxylic acid cycle; OXPHOS, oxidative phosphorylation.

**Figure 2:**
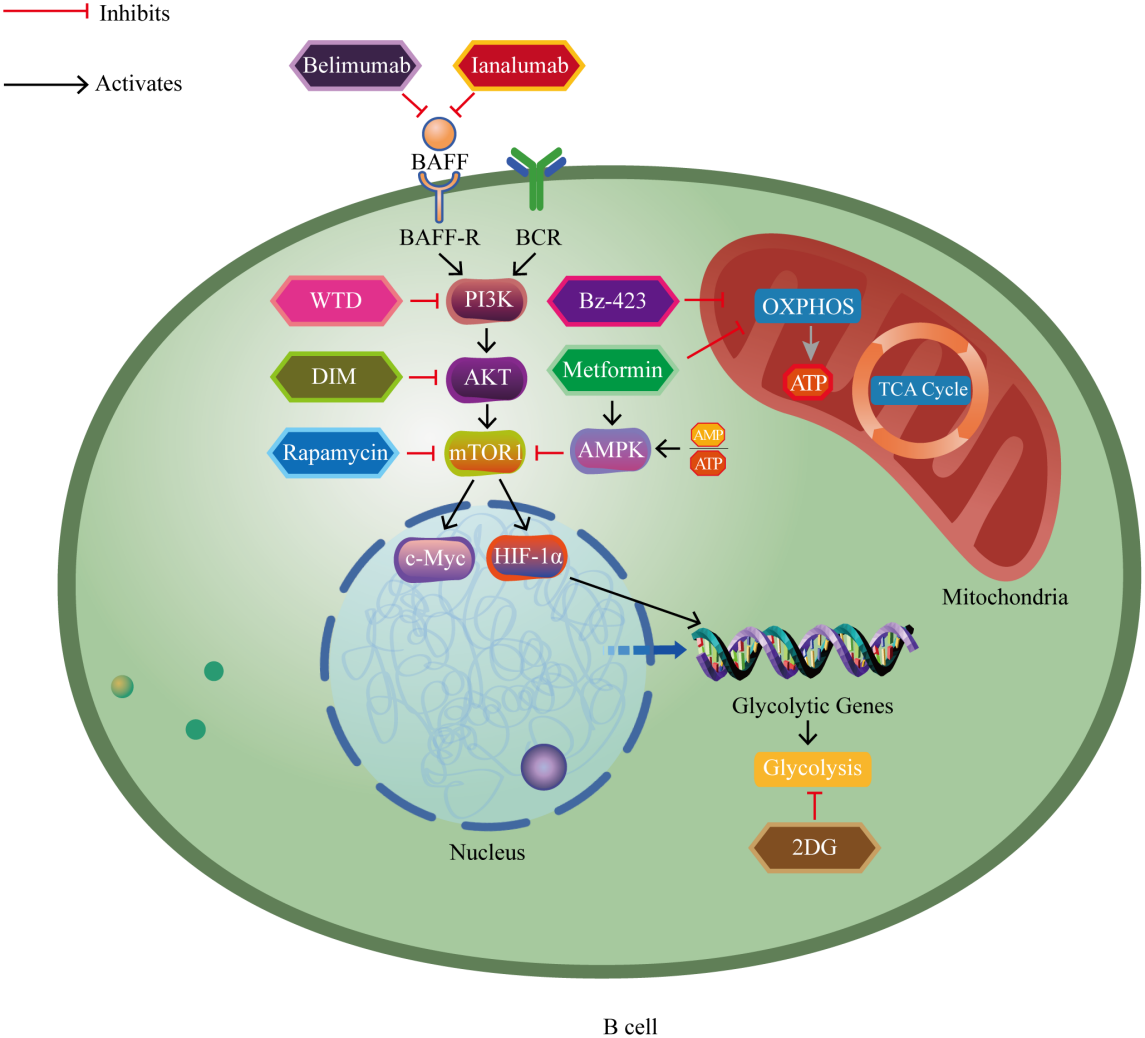
The impact of major metabolic drugs for treating autoimmune diseases on B cell metabolism pathways. Belimumab and ianalumab inhibit overexpression of BAFF and its activation-related downstream cascades. Rapamycin inhibits mTOR and its activation-related downstream cascades. Metformin activates AMPK, thereby inhibiting mTOR and its activation-related downstream cascades. 2DG inhibits glycolysis. WTD blocks the PI3K-AKT-mTOR-HIF-1α pathway and its activation-related downstream cascades. DIM inhibits the AKT/mTOR pathway and its activation-related downstream cascades. Metformin and Bz-423 inhibit oxidative phosphorylation. BAFF, B cell activating factor; BAFF-R, B cell activating factor receptor; BCR, B cell receptor; PI3K, phosphoinositide 3-kinase; AKT, protein kinase B; mTOR1, mammalian target of rapamycin complex 1; HIF-1α, hypoxia-inducible factor 1-alpha; c-Myc, cellular myelocytomatosis oncogene; AMP, adenosine monophosphate; ATP, adenosine triphosphate; AMPK, AMP-activated protein kinase; WTD, Wutou decoction; DIM, 3’3-Diindolylmethane; 2DG, 2-deoxy-D-glucose; Bz-423, 1,4-benzodiazepine; TCA cycle, tricarboxylic acid cycle; OXPHOS, oxidative phosphorylation.

Recent evidence has increasingly indicated the significant role of B lymphocytes in autoimmune disorders (5–8). Autoreactive B cells function to present self-derived peptides to autoreactive T cells. This interaction triggers T cell activation and promotes the generation of pathogenic autoantibodies from B cells (9). Progress in immunometabolism research has progressively recognized the substantial function of B lymphocyte metabolism modulation in autoimmune disorders (10, 11). B cells adjust their metabolic signaling pathways such as glycolysis and oxidative phosphorylation to fulfill the energy and biosynthetic requirements essential for their proliferation and division. The regulation and reprogramming of metabolic pathways provide the necessary metabolic support for B cell function and immune responses (12). However, imbalances in immunometabolism may lead to autoreactive B cells evading self-tolerance checkpoints, thereby inducing autoimmune diseases (13). Currently, research on B cell metabolism lags behind that of T cell metabolism, and further exploration of B cell metabolism represents a challenging yet promising area of study (14). In brief, we aim to provide a comprehensive conclusion of the latest research findings, focusing on the metabolic reprogramming of B cells in autoimmune disorders. We highlight key metabolic pathways and regulatory factors that manipulate B cell homeostasis, differentiation, and function (**Figure 1**), and also discuss the potential of metabolic interventions as a treatment for autoimmune disorders (**Figure 2**).

## Systemic lupus erythematosus

### Glycolysis

Systemic lupus erythematosus (SLE), a refractory and chronic autoimmune disorders, affects the skin, kidneys, and blood (15). B cell tolerance checkpoints function to limit the potential harm posed by autoreactive B cell repertoire and are related to important metabolic components. Dysfunction of these checkpoints is one of the mechanisms underlying the pathogenesis of SLE. For example, metabolic imbalances may compromise the function of these checkpoints, and heightened exposure to B cell activating factor (BAFF) proves a prominent mechanism bringing about tolerance defects. Individuals diagnosed with SLE exhibit significantly elevated BAFF levels in their serum compared to healthy individuals (16). Furthermore, individuals diagnosed with lupus nephritis (LN) had elevated levels of BAFF biological activity compared to individuals diagnosed with SLE but without LN. BAFF levels are positively related to disease activity. A study suggests that SLE patients receiving intensified treatment with high-dose glucocorticoids (GCs) exhibit a significant decrease levels of BAFF in their serum. Conversely, the serum BAFF levels increase when GC dosage is reduced (17). B cell activating factor (BAFF), a type II membrane-bound category protein, primarily promotes B cell activation and differentiation by combining with receptors on B cells: B cell activating factor receptor (BAFF-R), transmembrane activator and calcium modulator and cyclophilin ligand interactor (TACI), and B cell maturation antigen (BCMA) (18). The ability of BAFF in the maintenance and enhancing of B lymphocytes immune reaction is of great importance. However, increased levels of BAFF in SLE perhaps lead to abnormal B lymphocytes activation and autoimmune dysregulation (19, 20). Transgenic mice overexpressing the BAFF gene exhibit various features, including polyclonal hypergammaglobulinemia, heightened levels of anti-double-stranded DNA (anti-dsDNA) autoantibodies and deposition of immunoglobulins in the kidneys (21–23). This phenomenon is attributed to excessive BAFF-mediated rescue of autoreactive B cells, promoting their survival and proliferation (24). Research indicates that BAFF treatment leads to an augmentation in protein levels and the expression of genes that facilitate glycolytic metabolism (25). Compared to normal B cells after stimulated by lipopolysaccharide (LPS), B cells derived from BAFF transgenic mice exhibited increased glycolytic level (26). BAFF holds a prominent position in autoimmune disorders, particularly via disrupting the B cells glucose homeostasis. Prolonged exposure to BAFF activates the Erk1/2 pathway, leading to the promotion of glycolysis, enhanced cellular proliferation, and the rescue of autoreactive B cells. BAFF’s connection with receptors activates the PI3K/AKT/mTOR pathway. It is supported by research that the aforementioned pathway displays heightened activity within the B cells of individuals affected by SLE. Moreover, it demonstrates elevated expression levels in a manner that is influenced by both age and the dosage of lupus susceptibility genes (27). Bisphenol A (BPA), a commonly found environmental chemical, disrupts endocrine function and exhibits estrogenic properties. Exposure to BPA leads to upregulation of the PI3K/AKT/mTOR signaling pathway, which induces the onset of SLE (28). The signaling pathway known as phosphoinositide 3-kinase (PI3K) holds significant importance in the processes of B cell activation and maturation. PI3K is activated upon stimulation through surface signaling receptors such as BAFF-R, toll-like receptors (TLRs), IL-4 receptors (IL-4R), CD40. This leads to the recruitment of AKT to the cell membrane, where it gets phosphorylated by phosphoinositide-dependent kinase 1 (PDK1) and the mammalian target of rapamycin complex 2 (mTORC2). The PI3K signaling pathway regulates metabolism by targeting metabolic genes such as hexokinase 2 (HK2), phosphofructokinase 2 (PFK2), and cellular myelocytomatosis oncogene (c-Myc) (29). AKT, a serine/threonine kinase, regulates over 100 known substrates, thereby collectively controlling cellular growth, metabolism, and cell cycle. Its downstream signaling nodes include mammalian target of rapamycin complex 1 (mTORC1) and glycogen synthase kinase 3 (GSK3) (30). Studies have confirmed a remarkable increase in AKT in unstimulated B cells in a SLE mouse model (27). mTOR, a serine/threonine kinase, executes cellular functions including survival, differentiation, and metabolism by forming two complexes defined as mTOR complex 1 and 2 (mTORC1 and mTORC2) (31). Although these complexes share the “ancillary” proteins of mTOR, they regulate distinct functions and can be distinguished by the distinct involvement of scaffold proteins Raptor and Rictor (32). B cells in SLE display higher mTORC1 activity, promoting plasmablasts generation (33). The overactivated B cells upregulate the expression of mTORC1, which facilitates B lymphocyte proliferation and survival. mTORC1 further enhances glycolysis, thereby allowing autoreactive B cells to evade metabolic restrictions (13, 34).

Two crucial inhibitory regulators of the PI3K/AKT/mTOR pathway, the phosphatase and tensin homolog (PTEN) and AMP-activated protein kinase (AMPK), play considerable roles in maintaining B cell homeostasis. B cells derived from individuals with SLE demonstrate diminished PTEN expression compared with B cells from healthy individuals. Furthermore, the expression level of PTEN is inversely correlated with disease activity (35). Phosphatase and tensin homolog (PTEN) is critical depressor of cellular growth and proliferation, and it also exerts a significant influence on the regulation of immune system balance. PTEN functions to negatively regulate the PI3K/AKT/mTOR pathway via its bioactivity of lipid phosphatase (36). Specifically, PTEN directly counteracts PI3K, thereby preventing B cells from becoming excessively activated (37). AMP-activated protein kinase (AMPK) functions to detect the AMP/ATP ratio within the cell, initiating the activation of AMPK while concurrently inhibiting the mTOR signaling pathway when the ratio surpasses a certain threshold (38). AMPK and mTOR signaling pathways are in a dynamic balance during the development of B cells (39).

Ping Xie’s research provides compelling evidence for the indispensable contribution of TRAF3 in maintaining the homeostasis of B lymphocytes. Specifically, TRAF3-deficient B cell in mice leads to significant expansion of peripheral B cells, resulting in elevated levels of immunoglobulinemia. This condition is accompanied by enhanced T-independent antibody responses, as well as splenomegaly and lymphadenopathy, which are hallmark features of autoimmune disorders (40). A recent study has revealed that TRAF3 knockdown attenuates lupus nephritis symptoms in mice, including urinary protein excretion and renal inflammation, implying that targeting TRAF3 presents a promising avenue for therapeutic intervention in SLE (41). The above researches show that TRAF3 loss in B cells induce autoimmune diseases under physiological conditions, while TRAF3 knockdown alleviates SLE symptoms under pathological conditions. Tumor necrosis factor receptor (TNFR)-associated factor 3 (TRAF3), a cytoplasmic adapter protein, primarily regulating B cell signaling pathways (42). TRAF3 deficiency induces the overactivation of B cells by intrinsically activating multiple pro-inflammatory pathways which increases the risk of autoimmune disorders (43). Recent studies have demonstrated that TRAF3 ablation in B cell induces glycolysis (44). TRAF3 deficiency promotes the glucose metabolism of B cells, characterized by the upregulation of glucose transport 1 (GLUT1) and hexokinase 2 (HK2).

The dysregulated activation of the AKT/GSK3β signaling pathway in the lymphocytes of individuals with SLE makes a profound contribution to the development of the disease (45). Glycogen synthase kinase-3 (GSK3), a widely expressed kinase, is known to have more than 100 substrates, impacting cellular differentiation, proliferation, survival, and transformation (46). GSK3 exerts a restraining effect on B cell activation through the suppression of metabolic activity induced by CD40 and IL-4. Furthermore, GSK3 downregulates c-Myc-dependent glycolysis regulating energy production (47). Studies have reported that GSK3 inactivation synergistically induces the transcription factors Foxo1 and c-Myc, promoting the formation of plasma cells under CD40L and IL-21 stimulation (48).

Belimumab, a BAFF inhibitor, has received approval from the FDA as the sole targeted medication that has demonstrated effective outcomes in the treatment of SLE (49). Belimumab, a recombinant human immunoglobulin G (IgG)1-l monoclonal antibody, significantly reduces disease activity, decreases the use of steroids, and improves health-related quality of life (50). Belimumab specifically targets and binds to soluble BAFF, effectively counteracting its biological effects by obstructing its contact with BAFF-R, TACI, and BCMA receptors. According to a phase III randomized controlled trial study, belimumab is a promising treatment option for lupus nephritis in East Asian populations due to its efficacy and safety (51). A randomized phase III/IV clinical trial conducted over a period of 52 weeks, demonstrates that belimumab treatment yields greater improvement in disease severity compared to placebo in African-American patients with SLE (52). A retrospective observational study found that belimumab exhibits efficacy as a viable treatment option for individuals undergoing maintenance therapy for SLE (53). A retrospective, single-center study revealed that belimumab administration in pediatric patients with childhood-onset SLE has demonstrated positive effects on laboratory parameters, disease activity reduction, and a potential decrease in glucocorticoid dosage requirements. Furthermore, belimumab has exhibited a favorable safety record (54). Povetacicept, an enhanced dual APRIL/BAFF antagonist, demonstrates remarkable improvements in multiple disease indicators in a murine lupus model (55). Compared to conventional treatment, the combination of belimumab and low-dose intravenous cyclophosphamide (CYC) therapy presents a remarkable restoration of T and B cell equilibrium and significantly reduces disease activity scores among patients diagnosed with SLE. This treatment also significantly reduces adverse events such as infections (56).

Rapamycin, a promising therapeutic approach for SLE, functions as an inhibitor of mechanistic Target of Rapamycin Complex 1 (mTORC1), based on a clinical trial (57). During a 12-month period of administering rapamycin, the condition of SLE patients showed a steady amelioration in disease activity (58). Another study also demonstrated that rapamycin can attenuate pathological changes and reduce anti-dsDNA antibody titers in a mouse model of SLE (59). Rapamycin inhibits mTORC1, which reduces cellular uptake of glucose and glutamine, and inhibits glycolysis and glutamine degradation (27).

Treatment with 2-deoxy-D-glucose (2DG) to inhibit glycolysis greatly reduced the production of T cell-independent (TI) antigen-specific antibodies (60). *In vivo* treatment with metformin and 2DG reversed disease biomarkers and improved autoimmune symptoms in lupus-prone mice (61). Metformin is known to activate AMPK, leading to the downregulation of mTORC1 activity, which induces ATP breakdown pathways like glycolysis and fatty acid oxidation (FAO), while inhibiting ATP synthesis pathways including gluconeogenesis and lipogenesis. Metformin will be discussed later in the oxidative phosphorylation section.

### Oxidative phosphorylation

B cell metabolism like glycolysis and mitochondrial respiration were both increased in lupus-prone mice. As mentioned earlier, the increased BAFF not only upregulates glycolysis but also promotes the influx of pyruvate into the mitochondria, enhancing oxidative metabolism (26, 62) and inducing an elevation in mitochondrial membrane potential (25). BAFF activates downstream mTORC1, promoting an increase in mitochondrial mass and OXPHOS, thereby enabling the autoreactive B cells to evade energy crises (13, 34). BAFF inhibitor belimumab also has the ability to inhibit OXPHOS (20, 63).

The deficiency of TRAF3 also induces B cell hyperactivation by enhancing mitochondrial respiration, thereby increasing the risk of autoimmune diseases (43). TRAF3 ablation in B cell induces glucose uptake and oxidative phosphorylation enhancement, while mitochondrial mass and reactive oxygen species (ROS) production remain unchanged (44). Furthermore, downregulation of GSK3 attenuates oxidative respiration, thereby reducing ROS-mediated cellular toxicity (47).

Metformin is a commonly medication for managing type 2 diabetes. It reduces blood glucose levels through multiple mechanisms, including enhancing cellular glucose uptake and utilization, suppressing hepatic gluconeogenesis, and improving insulin sensitivity (64). Metformin has been found to inhibit OXPHOS. Metformin ameliorates renal dysfunction in MRL/lpr lupus-prone mice, as proved by reductions in urinary protein and blood urea nitrogen levels. Furthermore, metformin decreases IgG and complement C3 deposition, and attenuates systemic and renal inflammation in this murine model (65). Metformin alleviates the autoimmune phenotype including kidney inflammation in lupus mice via restricting B cell transformation into plasma cells (PC) and germinal centers (GC) (66). A *post-hoc* analysis of metformin adjunctive therapy in Chinese patients diagnosed with mild to moderate SLE revealed that metformin improves therapy outcomes in SLE patients (67). Bz-423 is also a mitochondrial metabolism inhibitor. It targets mitochondrial F1F0ATP synthase, leading to increased ROS levels, which in turn induces apoptosis in autoreactive cells, thereby suppressing disease manifestations in lupus-prone mice (68–70).

### Rheumatoid arthritis

Rheumatoid arthritis (RA), an intractable autoimmune condition, affects the hands, wrists, knees, elbows, and ankles, resulting in joint pain, swelling, stiffness, and functional impairment (71). Multiple investigations have consistently demonstrated that individuals diagnosed with RA exhibit increased levels of BAFF in their serum compared to healthy control (72–74). There exists a positive relationship between disease activity and serum BAFF levels (73). Belimumab is also used to treat RA (75). A Phase II randomized, double-blind clinical trial demonstrated the curative effect of belimumab in RA patients who have failed prior treatments, and it is generally well-tolerated (76). Increased mTOR activity has been observed in RA, and inhibiting mTOR has demonstrated moderate effectiveness in decreasing joint inflammation among individuals with RA (77, 78). Wutou Decoction (WTD) restrains angiogenesis by intercepting the PI3K-AKT-mTOR-HIF-1α pathway and improves RA symptoms in collagen-induced arthritis (CIA) model rats (79). 3’3-Diindolylmethane (DIM) restrains the generation and expansion of RA fibroblast-like synoviocytes (RA-FLSs) via inhibiting the AKT/mTOR pathway, reduces TNF-α-induced cytokines, and alleviates the severity of knee joint arthritis, preventing inflammation and knee joint destruction (80). A prospective randomized controlled study showed that metformin exhibited a notable improvement in the inflammatory response, disease severity, and living quality among individuals diagnosed with RA (81). Methotrexate (MTX) is a folate analogue compound that inhibits the folate metabolism enzyme and blocks a carbon transfer reaction necessary for *de novo* nucleotide synthesis. It has been used for the treatment of RA for over four decades (82).

### Type 1 diabetes

Type 1 diabetes (T1D), an intractable disease primarily driven by T cells, where pancreatic beta cells are targeted and destroyed by these autoreactive T cells (83, 84). Currently, there is a prevailing belief that B lymphocytes function indispensably in T1D via expressing co-stimulatory molecules and presenting antigens, which ultimately contributes to the activation of autoreactive T lymphocytes (85). Increasing evidence supports the notion that B cell development, differentiation, function, and metabolism have significant impacts on the development of T1D (86–89). Nonobese diabetic (NOD) mice serve as an exceptional model for investigating type 1 diabetes in human (90). Studies have shown that NOD mice with B cell defects are protected from T1D development (91). NOD mice lacking toll-like receptor 7 (TLR7) exhibit impaired B cell antigen presentation and antibody secretion, which inhibits activation of diabetogenic and cytotoxic T cells, and prolongs the time before T1D manifests (92). Furthermore, T1D patients have reduced PTEN expression in B cells, potentially resulting in an increased B cell autoreactivity (93). Rituximab, a selective anti-CD20 monoclonal antibody, has proved to deplete B lymphocytes effectively (94). A phase II study demonstrated that rituximab treatment in recently diagnosed T1D patients significantly lowers glycated hemoglobin levels, elevates C-peptide levels, and reduces exogenous insulin requirement (95).

### Other autoimmune diseases

Primary Sjögren’s Syndrome (pSS) is distinguished by immune attack on the exocrine glands. This condition results in diminished or lacking secretions, causing dryness in the mouth and eyes (96). Immunoglobulin A (IgA) nephropathy is distinguished by the accumulation of abundant IgA proteins in the glomeruli of the kidney (97). In both pSS and IgA nephropathy, the serum levels of BAFF are elevated compared to healthy levels (72, 98). In pSS patients, an increase in the AMPK/mTORC1 activity ratio in B cells of salivary glands inhibits synthetic metabolism and increases susceptibility to oxidative stress (99). Belimumab and ianalumab, two monoclonal antibodies targeting BAFF, have been developed as potential therapies for pSS. A phase II study proved that belimumab had some curative effect and safety in the therapy of pSS (100). In a double-blind, placebo-controlled, phase II study, ianalumab was found to effectively deplete B cells and provide therapeutic benefits to pSS patients without any significant side effects (101).

### Conclusion and perspective

While targeting the metabolism of autoreactive B cells is a prospective strategy for the management of autoimmune disorders, there remain several challenges to be addressed in the future. Firstly, the current understanding of the metabolic reprogramming of B cell function under physiological and pathological conditions is still incomplete and disputable, limiting the ability to precisely target specific metabolic pathways for the treatment of certain autoimmune disorders. For instance, it is generally believed that resting B lymphocytes predominantly rely on mitochondrial oxidative phosphorylation for energy production, while activated B lymphocytes tend to favor glycolysis as their metabolic pathway to rapidly provide energy for proliferation, differentiation, and other functions (102–104). However, several studies have also demonstrated that stimulated B cells do not heavily rely on glycolysis. B cell functions were not impacted by glucose restriction. On contradiction, B cell growth and differentiation were significantly hindered by either the inhibition of OXPHOS or a restriction in glutamine (105). Additionally, research has revealed that GC B cells consume more glucose and exhibit greater sensitivity to glycolysis inhibition compared to naive B cells (47, 106). Contrary to this, studies have demonstrated that GC B cells utilize glycolysis minimally, primarily relying on fatty acid oxidation to generate energy (107). As is widely known, proliferating B lymphocytes heavily rely on lactate dehydrogenase A (LDHA) for aerobic glycolysis. Nevertheless, a recent study has revealed that in naive B cells, the deletion of the glycolytic enzyme LDHA leads to impaired germinal center formation and antibody production. The impact of LDHA knockout on activated B cells is relatively minor (108). In conclusion, there is a relatively limited and controversial amount of research data regarding B cell metabolism currently. Therefore, a substantial amount of further research is required to elucidate the role of B cell metabolism in autoimmune diseases.

Secondly, most experimental studies on B cell metabolism have focused on mice rather than humans. Although valuable insights have been gained through the use of genetically modified mice, it is necessary to consider the distinction between mouse and human immune systems and metabolic pathways. For instance, B cells upregulate GLUT1 expression to increase glucose uptake in mice. However, in humans, B cell glucose uptake relies on other transporters due to relatively lower expression of GLUT1 in these cells (102, 109). Therefore, to ensure the successful application of the research findings from mice to humans, further exploration and examination are indisputably necessary.

Thirdly, traditional therapeutic approaches for autoimmune diseases involve the use of glucocorticoids and immunosuppressive agents, which may lead to various side effects such as infections, osteoporosis, and hypertension (110). Accordingly, there is a continuous search for personalized drugs with high safety profiles. Combining metabolic modulators or biologics with existing immunosuppressive agents offers a potential solution to reduce the dosage of traditional medications, thereby lowering the incidence of adverse reactions. Furthermore, there are significant individual differences in drug tolerance and therapeutic efficacy among autoimmune disease patients receiving targeted metabolic therapy. Thus, employing metabolomics and other techniques to pinpoint specific biomarkers for precise treatment may be necessary. Finally, with the rapid progress of new technologies, there is a compelling need to expand the utilization of innovative methodologies such as single-cell metabolomics with mass spectrometry and spatial transcriptomics (111) in autoimmune disease research. Traditional metabolomics studies focus on the overall metabolism of B cells or large B cell subpopulations. However, leveraging single-cell metabolomics provides a promising avenue to uncover novel, functionally distinct subsets with unique metabolic patterns and gain comprehensive insights into metabolic events (112). Understanding the spatial dynamics of B cells in physiological or disease states, including their metabolic microenvironment and interactions with other leukocyte populations, is of utmost importance for comprehending B cell functionality.

In this review, we provide a concise overview of the metabolic alterations observed in B cells in autoimmune diseases, emphasizing critical signaling pathways and molecules. Targeting and restricting B cell metabolism have emerged as potential therapeutic strategies for treating autoimmune diseases. However, the influence of B cell metabolism on autoimmune diseases warrants further investigation. Taken together, the modulation of B cell metabolism may represent a promising stratagem and viable avenue for future autoimmune disorder treatments.

## Author contributions

BZ, ZZ and JL conceived and designed the structure of the literature review. JL conducted literature research, drafted the manuscript and created the figures. MZ, JH and WL commented and revised the manuscript. All authors contributed to the article and approved the submitted version.
